# Measuring attitudes towards voluntary childlessness: Indicators in European comparative surveys

**DOI:** 10.1371/journal.pone.0319081

**Published:** 2025-03-19

**Authors:** Ivett Szalma, Marieke Heers, Maria Letizia Tanturri

**Affiliations:** 1 Institute for Sociology, HUN-REN Centre for Social Sciences, Institute for Sociology, Budapest, Hungary; 2 Swiss Centre of Expertise in the Social Sciences (FORS), c/o University of Lausanne, Lausanne, Vaud, Switzerland; 3 Department of Statistical Sciences, University of Padua, Padua, Italy; University of Ghana School of Physical and Mathematical Sciences, GHANA

## Abstract

The prevalence of voluntary childlessness is rising in Europe, likely accompanied by growing social acceptance. However, more evidence is needed on how to measure this acceptance in comparative surveys. This study examines two dimensions of voluntary childlessness: prescriptive and proscriptive. The aim of this study is to show how the two dimensions differ in measuring voluntary childlessness and to highlight the factors that shape these attitudes. Findings reveal that socio-demographic variables relate differently to the two dimensions of the acceptance of voluntary childlessness for men and women. At the macro level, lower gender inequality predicts higher acceptance of voluntary childlessness across both dimensions. Conversely, higher childlessness rates are associated with favourable attitudes only in the prescriptive dimension, while country-level religiosity does not predict either dimension. The study underscores the importance of distinguishing between dimensions of voluntary childlessness, as different factors shape their acceptance. Additionally, no differences emerge in attitudes toward voluntary childlessness for males and females.

## Introduction

Across Europe, it is widely believed that parenthood is essential for a meaningful and fulfilling life [1]. Traditionally, societal norms and cultural values have favoured reproduction and parenthood, while childlessness has been stigmatized, particularly for women [2]. However, since the 1960s, significant cultural and structural transformations have reshaped the concepts of adulthood, family, and parenthood. These decades have experienced major ideological and cultural shifts that emphasize self-fulfilment, personal choice, development, and freedom. As a result, having children has become one of many potential life choices rather than an obligatory societal expectation [3]. Some researchers suggest that the secularization process, marked by a decline in the influence of religious beliefs and practices and the loosening of rigid social norms, has empowered women to make authentic choices about their lives, including decisions regarding family and career [4]. Others argue that the choice to remain childless means a significant step in women’s liberation from traditional gender roles [5].

Since the 1970s, the availability of reliable contraceptives has further enabled women to decide whether or not to have children while remaining sexually active. This technological advancement not only empowered women to remain sexually active without becoming mothers but also contributed to the growing visibility and prevalence of voluntary childlessness [6]. As childlessness becomes more widespread in Western societies [7], the societal expectation to have children diminishes, reinforcing this trend [8].

Numerous studies have examined voluntary childlessness [8–11], and there are related questions in national and international surveys. However, ambiguity often arises when respondents are prompted to articulate their attitudes towards remaining childless, as it is unclear what specific circumstances they consider. Research indicates that attitudes towards voluntary childlessness vary across different countries and between genders [9–12]. Some studies suggest stronger societal expectations for women to become parents [13,14], while others find more favourable attitudes towards male childlessness [11].

In a recent study, de La Rochebrochard and Rozée [15] explored how different survey designs can lead to contradictory results. While their study provided valuable insights, it overlooked the potential role of item wording—a critical factor that might shape responses and capture different dimensions of voluntary childlessness. It is theoretically plausible that both survey design and item wording influence results, as different wordings might capture various dimensions of attitudes towards childlessness.

The aim of this study is twofold: first, to examine the factors shaping attitudes towards voluntary childlessness, and second, to compare two measures to determine the extent to which they assess the same dimension of acceptance of voluntary childlessness. We begin by reviewing the questions used in previous research, focusing on those in international comparative surveys. To assess these measures, we replicate the analysis using data from two surveys designed to evaluate attitudes towards voluntary childlessness. If our analyses produce consistent results, both measures can be considered reliable and valid for this construct. Empirically examining attitudes towards voluntarily childless populations is essential because it helps understand how these attitudes change over time and across contexts, especially as the proportion of voluntarily childless individuals is expected to rise. Furthermore, it highlights the implications of using diverse measures of voluntary childlessness, which is vital for developing valid survey instruments.

## Theoretical framework and literature review on voluntary childlessness

### The various forms of childlessness

Childlessness can take various forms, each of which plays a significant role in understanding the societal norms and expectations surrounding parenthood. Lifetime or permanent childlessness refers to individuals who have not had biological or adopted children by the end of their reproductive years. This group is heterogeneous, with diverse experiences across temporal, motivational, and normative aspects [16]. Social parenting, where individuals without biological or legal ties still engage in parental-like roles, often goes unrecognized in fertility and family life surveys [17].

Childlessness, as a non-event, challenges understanding the reasons behind the decision to remain childless. In many circumstances, the entire process leading to childlessness might be blurred: in some cases, the paths leading to childlessness are part of a rational choice process, adopted consciously to reject parenthood. However, it is frequently caused by a continuous sequence of postponements in childbearing [18]. Literature identifies individuals who aspire to have children but postpone parenthood endlessly until they abandon it or it becomes too late to conceive. In this scenario, childlessness is not an intentional decision. Furthermore, childlessness can be explained by medical conditions.

Childlessness is often categorized as either voluntary or involuntary [16]. Voluntary childlessness involves individuals who choose not to have children, while involuntary childlessness includes those unable to do so due to external factors like relationship issues, medical problems, or financial concerns. This binary classification overlooks those who intend to have children but delay until biological limitations or socially accepted timeframes prevent it [16]. Furthermore, it does not address the situation of same-sex couples who wish to have children but face societal barriers, such as legal or social discrimination, in certain societies [19].

### Prescriptive vs. proscriptive attitudes towards voluntary childlessness

Shapiro’s review [20] of 30 years of research on voluntary childlessness highlighted the underexplored nature of the topic. The review identified four key areas of debate: 1) who chooses to be childless; 2) why individuals choose voluntary childlessness; 3) the stigmatization of voluntary childlessness; and 4) the consequences of voluntary childlessness [20]. While the first two research areas focus on the characteristics of the voluntarily childlessness population, our study focuses on the latter two areas: attitudes toward voluntary childlessness.

Attitudes toward voluntary childlessness can be categorized into two distinct types: prescriptive and proscriptive attitudes. Prescriptive attitudes reflect the social expectation and desirability of parenthood. These attitudes convey the belief that having children is an essential part of fulfilment and social responsibility. According to Koropeckyj-Cox and Pendell [21,22], prescriptive norms emphasize the expectation that individuals, especially women, are expected to become parents. For instance, the European Social Survey (ESS) asked respondents: *‘How much do you approve or disapprove if a woman/man chooses never to have children*?*’* This item reflects prescriptive attitudes, as it highlights societal approval or disapproval of the decision to remain childless. This is linked to stigma, as societal attitudes that disapprove of voluntary childlessness may lead to various forms of social punishment, such as stigma [20].

In contrast, proscriptive attitudes focus on the perceived disadvantages of childlessness. These attitudes are less about societal expectations and more about the negative evaluation of voluntary childlessness. For example, the European Values Study (EVS) asked participants whether *‘a woman/man has to have children in order to be fulfilled*.*’* This is a proscriptive item, as it suggests that childlessness may be viewed negatively, as something potentially leading to an unfulfilled life. The focus of proscriptive items is on what is perceived as undesirable or lacking when someone remains childless by choice [20].

These two types of attitudes are important for the societal dynamics surrounding voluntary childlessness. Prescriptive attitudes highlight the expectation for individuals to become parents, while proscriptive attitudes underscore the negative social consequences of choosing to remain childless. Both attitudes can reinforce the stigmatization of voluntary childlessness, shaping how individuals are perceived in society. By analysing both prescriptive and proscriptive attitudes, we can better understand the complex societal factors that influence individuals’ decisions regarding voluntary childlessness, as well as the stigma that may accompany such choices.

### Theories on voluntary childlessness

In order to explain low fertility and the rising prevalence of childlessness, demographers have relied on different theories including New Home Economics [23], Gender Revolution [24,25], and Second Demographic Transition Theory (SDT) [26]. These frameworks emphasize different mechanisms at both individual and societal levels, shedding light on attitudes toward voluntary childlessness.

The New Home Economics framework underscores the increasing opportunity costs of childbearing as women participate in the labour market [10,23,27]. Higher opportunity costs lead women to be more supportive of voluntary childlessness than men [11,15,22]. Similarly, higher education is a key predictor of more accepting attitudes toward childlessness within this framework, as individuals with greater human capital often prioritize professional and leisure pursuits over traditional family roles [11,15,21,22]. Employment status also influences attitudes, with employed individuals generally holding less traditional views on childlessness.

Consistent with the New Home Economics, Eicher and coauthors [9] hypothesized that in countries where childcare availability is broader, attitudes toward childlessness would be more accepting, as these conditions allow mothers to combine childbearing with employment. However, their findings revealed no significant macro-level impact of childcare availability or maternal employment on attitudes toward childlessness.

The Gender Revolution theory focuses on the transition from the male-breadwinner model to new egalitarian structures based on dual-earner couples. It predicts negative effects on fertility due to the potential inconsistency between gender equity—defined as the perceived fairness of gender roles—and actual gender equality. McDonald [24] suggested that an increased gender equality in institutions like paid jobs and education, if it is not accompanied by gender equality in the household – with men sharing domestic and care chores – may depress fertility levels. Empirical evidence supports this theory, linking higher societal gender equality—measured by the Gender Inequality Index—to more accepting attitudes toward childlessness [9,11,28].

The Second Demographic Transition theory focuses on emerging post-materialistic values related to self-realization and autonomy that progressively replace religious norms, influencing new generations’ family ideals and reproductive behaviour [29]. Secularization is a key component, as non-religious individuals are more likely to reject traditional family norms and support childfree lifestyles [29–31]. Studies show that religious individuals are more likely to disapprove of voluntary childlessness [10,21,22]. Additionally, younger generations, shaped by post-materialistic values, tend to prioritize autonomy and self-realization over traditional family roles, leading to more favourable attitudes toward voluntary childlessness [11,15,21,32].

Marital status also significantly influences attitudes. Individuals in traditional partnerships, such as marriage, or those who are widowed often hold more conventional views, whereas those in less traditional arrangements, such as cohabitation, exhibit greater acceptance of childfree lifestyles [11,32]. Higher rates of childlessness further reflect SDT progress, as societies with greater acceptance of male and female voluntary childlessness are typically more advanced along this demographic transition.

While the SDT theory emphasizes the connection between secularization and declining fertility, no research to date has examined how macro-level religiosity influences societal acceptance of voluntary childlessness. Addressing this research gap could provide deeper insights into how cultural and institutional factors shape the interplay between secularization and family norms.

## Measurement of attitudes towards voluntary childlessness

In this section, we present an overview of the measurements of attitudes towards voluntary childlessness previously used in international surveys, with a focus on Europe, and discusses the empirical insights derived from these instruments. So far, three European international surveys have measured attitudes towards voluntary childlessness.

The first international survey which included questions regarding attitudes towards voluntary childlessness is the International Social Survey Programme (ISSP) where the following item was used in 1988: *‘People who have never had children lead empty lives.’* This item was repeated in 1994 and 2002, offering a longitudinal perspective on changes in attitudes. However, because the ISSP focuses on perceptions of the childless lifestyle rather than specifically measuring voluntary childlessness, it is excluded from this analysis.

Two subsequent surveys addressed attitudes toward voluntary childlessness more explicitly and distinguished between responses regarding males and females. The European Values Study (EVS), in its 2001 and 2008 waves, included items asking: *‘Do you think that a woman/man has to have children in order to be fulfilled, or is this not necessary?*’** The response categories for women were binary (‘needs children’ vs. ‘not necessary’), whereas for men, a five-point Likert scale was used, ranging from ‘strongly agree’ to ‘strongly disagree.’

The European Social Survey (ESS) conducted in 2018 focused on a different aspect of attitudes towards voluntary childlessness by asking the following question: *‘How much do you approve or disapprove if a woman/man chooses never to have children?’* A split-sample design was applied, and respondents answered only one version of the item: half of them were surveyed concerning the norm for women (but not for men) and half concerning the norm for men (but not for women).

### Attitudes towards male and female voluntary childlessness

Previous research has often overlooked significant differences in attitudes towards voluntary childlessness between men and women. However, some studies have produced conflicting results regarding male and female attitudes towards childlessness. Qualitative research has found that voluntary childlessness carries more stigma for women than for men [33,34]. At the same time, Rijken and Merz [11], using ESS data, observed that society tends to be less disapproving of voluntary childlessness in men than in women. They argued that this discrepancy arises from the greater personal and professional burdens faced by women as a result of parenthood, whereas men’s lives are typically less impacted. This discrepancy is often referred to as “double standards”, a term that originates from the theory of status differences. More specifically, the term refers to how behaviours are perceived and evaluated differently based on an individual’s social category, such as gender or ethnicity. This cognitive bias often results in harsher judgment for those in lower status groups, while individuals in higher status groups tend to be evaluated more leniently. Double standards are also reflected in the levels of gender equality in countries: in countries with greater gender equality, it is more accepted for women to prioritize their careers over motherhood than in countries with lower gender equality.

However, de La Rochebrochard and Rozée [15] argue that the double standard cannot be accurately measured using data from the European Social Survey, as the ESS’s split-sample design only asks each respondent about either male or female norms, but not both. This design flaw prevents an accurate assessment of double standards, as it fails to capture responses from all participants on both items. To properly compare attitudes towards male and female voluntary childlessness, which is essential for identifying double standards, a measurement is needed that covers both genders and offers identical response options.

## Data and method

### Data

We utilized data from both the European Values Study [35] and the European Social Survey [36,37] to examine attitudes toward voluntary childlessness and conducted equivalent analyses. We examined the relationship of these variables with socio-demographic variables, that are available in both surveys. S1 Table in the Supporting information summarises the included database and variables in this study.

This approach allows us to investigate whether there are differences in the associations between socio-demographic determinants and attitudes towards voluntary childlessness when measured in different ways, using two variables across two surveys within the same set of countries. Our analysis includes 27 European countries that participated in both the EVS and the ESS: Austria, Belgium, Bulgaria, Croatia, Cyprus, Czech Republic, Denmark, Estonia, Finland, France, Germany, Hungary, Ireland, Iceland, Italy, Latvia, Lithuania, Poland, Portugal, Norway, the Netherlands, Slovakia, Slovenia, Spain, Sweden, Switzerland, and the United Kingdom.

The sample is intended to represent the adult population 18 years old and older. In the ESS individuals over 15 are surveyed; here, we restrict the sample to respondents who are 18 or older in order to harmonise the two datasets. Face-to-face interviews with a standardised questionnaire were conducted between 2018 and 2020 in the ESS. The EVS fieldwork was conducted between 2008 and 2010 through face-to-face interviews with standardised questionnaires. While attitudes generally change slowly, previous research suggests that attitudes towards childlessness may change rapidly [32]. Although the EVS 2008 data collection and the ESS 2006 data collection are relatively close in time, we ultimately decided to use the ESS 2018 data collection, as it allowed us to include a total of 27 countries in the analysis that are present in both datasets. The ESS 2006 data, only allows a comparison of 21 countries. For robustness, we also verified our calculations using the 2006 ESS data, which are detailed in the supplementary materials and provide results that are consistent with those presented here.

The research does not require an ethics statement because we analysed anonymised data.

#### Dependent variables: Prescriptive and proscriptive attitudes about voluntary childlessness.

The ESS includes one item with a 5-point Likert scale asking to what extent respondents approve or disapprove of the following statement: *‘How much do you approve or disapprove if a woman/man chooses never to have children?’* Response options ranged from 1 (strongly disapprove) to 5 (strongly approve). This item evaluates prescriptive attitudes towards childlessness. In order to limit the respondents' burden, a split-ballot design was implemented in the ESS to assess attitudes towards voluntary childlessness. Approximately fifty percent of the respondents were asked questions about male behaviour, while the remaining half were queried regarding female behaviour. In this paper, to emphasise the acceptance of voluntary childlessness, responses were grouped into two categories: positive (approve and strongly approve) and neutral or negative (disapprove and strongly disapprove) attitudes towards childlessness.

The following item was included in the EVS: *‘Do you think that a woman has to have children in order to be fulfilled or is this not necessary?’* The answer categories were: 1 = needs children; 2 = not necessary. Later in the survey the following question was asked*: ‘Do you agree or disagree with the following statement: A man has to have children in order to be fulfilled’.* The answer categories were measured on a 5-point scale, where 1 means agree strongly, and 5 means disagree strongly. These items are used to assess proscriptive societal norms about childlessness since they focus on the consequences of not having children.

For women, the categorization was straightforward: ‘needs children’ was coded as 0, and ‘not necessary’ was coded as 1. For men, we applied the same logic as in the ESS, since our goal was to measure the acceptance of voluntary childlessness. Therefore, we coded the ‘disagree’ and ‘strongly disagree’ response categories as 1, and ‘strongly agree’, ‘agree’, and ‘neither agree nor disagree’ as 0.

#### Independent individual-level variables.

Consistent with prior research, this study includes several variables as possible correlates of how individuals perceive family values. S2 Table in the Supporting information presents the independent variables included in the analysis.

The individual socio-demographic variables include gender (male = 1, female = 2), age group (divided into four categories: 18–30, 31–45, 46–60, >  60), level of educational attainment (categorized as low level education = 1, medium level education = 2, having at least a degree = 3), labour force status (classified as having a paid job = 1, not having a paid job = 2, retired = 3), partner status (grouped as not having a partner = 1, living with a partner but not married = 2, married = 3), having children (coded as having one or more = 1, no child = 0), and attendance of religious services (categorized as at least once a week, at least once a month, only on special holidays, less often, never). We used the attendance of religious services because this was the only item we could harmonize between the two datasets.

#### Independent country-level variables.

As seen in the theoretical framework, in addition to individual level characteristics also country level factors are likely to influence the dependent variable. Based on the literature on voluntary childlessness, we included three country-level variables. The childlessness rate, derived from EVS and ESS data, represents the percentage of respondents aged 40 + without biological children. Given that religious commitment appears to play a key role in shaping attitudes towards voluntary childlessness, we also constructed country-level variables based on both databases. To calculate the aggregated religiosity index at the country level, we averaged the sample’s individual responses to the question about religious service attendance for each country. This approach captures the general level of religiosity within each country, based on respondents’ self-reported frequency of attending religious services. The attendance categories in the analysis ranged from weekly attendance to never attending, and the mean value was computed to represent the overall religiosity of the population in each country.

Additionally, we included an external country-level indicator, the Gender Inequality Index (GII), which is not part of the ESS or EVS databases and was merged to the data. The GII, used in the Human Development Reports since 2010 [28], assesses gender inequality in each country by measuring women’s disadvantage across three dimensions: reproductive health, empowerment, and the labour market. GII values range from 0 (indicating equal outcomes for men and women) to 1 (indicating that women are disadvantaged in all measured dimensions). For the EVS data, we used the GII indicator corresponding to 2008, whereas for the ESS data, we employed the GII indicator for 2018.

### Analytical strategy

Three out of four dependent variables were measured on an ordered scale. One of the items, i.e. the EVS-item regarding women, was measured as a dummy variable. We used multilevel logistic regression models to study social attitudes towards voluntary childlessness, reducing Likert-scale categories into dummy variables: approval (approve/strongly approve) coded as 1, and non-approval (disapprove/neutral) coded as 0. For ‘*A man has to have children to be fulfilled,’* disagree/strongly disagree was coded as 1, and other responses as 0. Similarly*, ‘A woman has to have children to be fulfilled’* was coded as not needed = 1 and needed = 0.

To examine social attitudes towards voluntary childlessness, multilevel logistic regression models were used. The analyses account for both individual and contextual characteristics by modelling the data with a two-level structure, where individuals are nested within countries. Multilevel logistic regression also allows for estimating the proportion of total variation attributable to the aggregate level, helping to assess the strength of the contextual influence.

Model A included socio-demographic variables. Model B added the childlessness rate, Model C included the GII, and Model D added religious service attendance. We used design weights in all models. The Results section presents descriptive findings followed by logistic model results.

### Data limitations

Despite its high value, the data analysed in this study have some limitations. First of all, the data does not allow us to determine the exact scope and meaning of what the items measure. This aligns with the general limitation of survey research, where respondents cannot share the specific connotations, they associate with the given terms. Another limitation is that, while we harmonised the data and included the same independent variables and the same countries in the two databases, the EVS was run 10 years earlier than the ESS. We could have used the ESS 2006 data, as the difference with the EVS database would have been only two years, the results would not have brought significant changes (See the robustness check). Therefore, we decided to use the more recent data instead.

Finally, with the data analysed here, we cannot properly measure double standards because in the ESS only one of the items (either regarding women or regarding men) was asked from each respondent, while in the EVS the answer options were different in the case of the item regarding men and women.

## Results

### Descriptive results

Our findings indicate significant variations in the levels of acceptance towards male and female voluntary childlessness across Europe. S1 Fig in the Supporting information shows the percentage of respondents who accept female voluntary childlessness in each country in the two databases. We found that acceptance of female voluntary childlessness is lowest in the Central Eastern European (CEE) countries, and highest in the Netherlands and the Nordic countries. Meanwhile, Southern European countries, like other continental ones, are in the middle.

S2 Fig in the Supporting information presents the percentage of respondents who accept male voluntary childlessness in each country within the two databases. The northern countries display slightly greater variability in acceptance rates for male voluntary childlessness compared to females. Dutch respondents are the most accepting, followed by Norwegians. Belgians score highest on the prescriptive dimension. On the lower left side of S2 Fig the same CEE countries are positioned as in S1 Fig for female voluntary childlessness. Similarly, the Southern European countries again occupy a middle position.

Denmark presents a particularly interesting case, exhibiting notable differences across the two dimensions: while Danish individuals rank among the most accepting in the prescriptive dimension, their acceptance in the proscriptive dimension aligns more closely with the views of the CEE countries.

In order to determine whether the examined 27 European countries exhibit a significantly similar ranking in the two measures (ESS and EVS items), we conducted Kendall’s tau test. This nonparametric test assesses concordances and discordances in paired observations [38]. There is significant concordance among countries for both female and male measurements (Kendall’s score: female =  157, p = 0.013; male =  192, p = 0.001).

### Multivariate analysis

In this section, we present the results of multilevel logistic regressions. First, we discuss the results pertaining to female voluntary childlessness. Then, we present the results for male voluntary childlessness. Finally, we provide the results of the macro level indicators in all the models.

#### Results for individual level variables for items considering female voluntary childlessness.

Estimates of the multilevel logistic regressions predicting attitudes towards female voluntary childlessness based on the ESS data are shown in Tables 1 and 2.

**Table 1:** Results of the multilevel logistic regression predicting attitudes of female voluntary childlessness, ESS data 2018.

	Model A	Model B	Model C	Model D
Approve if woman chooses not to have children				
*Male*	*1.000*	*1.000*	*1.000*	*1.000*
Female	1.311***	1.312***	1.312***	1.311***
18-30	1.237**	1.237**	1.238**	1.237**
31-45	1.399***	1.399***	1.401***	1.400***
46-60	1.279***	1.279***	1.280***	1.279***
>60	*1.000*	*1.000*	*1.000*	*1.000*
Low (ISCED 0-2)	0.905**	0.904**	0.904**	0.904**
*Medium (ISCED 3-4)*	*1.000*	*1.000*	*1.000*	*1.000*
High (ISCED 5-6)	1.171***	1.171***	1.170***	1.171***
*Paid job*	*1.000*	*1.000*	*1.000*	*1.000*
Not in paid job	0.901*	0.901*	0.901*	0.901*
Retired	0.830**	0.830**	0.831**	0.830**
At least once a week	0.464***	0.464***	0.465***	0.464***
At least once a month	0.596***	0.596***	0.597***	0.597***
Only on special holy days	0.708***	0.708***	0.709***	0.709***
Less often	0.811***	0.811***	0.811***	0.811***
*Never*	*1.000*	*1.000*	*1.000*	*1.000*
Single	1.135**	1.135**	1.135**	1.135**
Cohabiting	1.248***	1.249***	1.247***	1.248***
*Married*	*1.000*	*1.000*	*1.000*	*1.000*
*Yes, have children*	*1.000*	*1.000*	*1.000*	*1.000*
Not having children	1.478***	1.478***	1.479***	1.479***
CHILDLESSNESS		1.064		
GII			0.872***	
ATTENDANCE				1.262
Constant	0.633	0.214*	2.968***	0.228
Constant (country)	4.523***	3.786***	1.824***	4.294***
ll likelihood	-12051.3	-12049.6	-12038.7	-12050.8
Wald Chi2	847.2	850.6	884.3	813.1
N (individuals/countries)	21954/27			

The standard errors are adjusted for clustering at the country-level. * p < 05; ** p < 0.01; *** p < 0.001.

**Table 2:** Results of the multilevel logistic regression predicting attitudes of female voluntary childlessness, EVS data 2008.

	Model A	Model B	Model C	Model D
A woman does not need children to be fulfilled				
*Male*	*1.000*	*1.000*	*1.000*	*1.000*
Female	1.243***	1.243***	1.243***	1.243***
18-30	1.210**	1.210**	1.210**	1.209**
31-45	1.516***	1.516***	1.516***	1.515***
46-60	1.384***	1.384***	1.384***	1.383***
>60	*1.000*	*1.000*	*1.000*	*1.000*
Low (ISCED 0-2)	0.783***	0.783***	0.783***	0.783***
Medium (ISCED 3-4)	*1.000*	*1.000*	*1.000*	*1.000*
High (ISCED 5-6)	1.134***	1.134***	1.134***	1.134***
*Paid job*	*1.000*	*1.000*	*1.000*	*1.000*
Not in paid job	0.939	0.939	0.939	0.939
Retired	0.921	0.921	0.922	0.921
At least once a week	0.573***	0.573***	0.574***	0.573***
At least once a month	0.652***	0.652***	0.652***	0.652***
Only on special holy days	0.691***	0.691***	0.691***	0.691***
Less often	0.782***	0.782***	0.782***	0.782***
*Never*	1	1	1	1
Single	1.006	1.006	1.006	1.006
Cohabiting	1.117*	1.117*	1117*	1.117*
*Married*	*1.000*	*1.000*	*1.000*	*1.000*
*Yes, have children*	*1.000*	*1.000*	*1.000*	*1.000*
Not having children	2.217***	2.217***	2.216***	2.217***
CHILDLESSNESS		1.126*		
GII			0.907**	
ATTENDANCE				0.316
Constant	0.944	0.329	17.697**	2.494
Constant (country)	4.653***	3.972	3.229***	4.405***
ll likelihood	-18979.3	-1897.9	-18975.7	-18978.8
Wald Chi2	1615.5	1618.1	1622.7	1616.4
N (individuals/countries)	34660/27			

The standard errors are adjusted for clustering at the country-level. *  p < 05; ** p < 0.01; *** p < 0.001.

Female respondents have more positive attitudes than male respondents concerning female voluntary childlessness for both measurements of attitudes towards female voluntary childlessness. There is a significant association between respondent’s age group and attitudes towards voluntary childlessness in both dimensions: compared to the oldest age group, those in the younger age groups have a more favourable attitude towards female voluntary childlessness. Regarding respondents’ educational level, both measurements show the same association: those who have the highest level of education have more favourable attitudes towards female voluntary childlessness for both measurements, while those with lower levels of education also reveal a significant, more rejecting attitude. We also found a significant association between employment status and attitudes towards female voluntary childlessness in both dimensions: pensioners are less likely to accept female voluntary childlessness. However, those who are not pensioners and do not have paid employment differ significantly from those in paid employment only in the prescriptive dimension: they are less likely to accept that a woman chooses not to have children.

In terms of religiosity, those attending religious services regularly report lower levels of acceptance compared to those who never visit religious services on both measurements of voluntary female childlessness. In both dimensions, those who live in a cohabiting relationship are significantly more likely to approve female voluntary childlessness than married respondents. While single individuals differed significantly from married ones only in the prescriptive dimension: they had a more favourable attitude towards female voluntary childlessness in the perspective dimension, but they did not differ from married individuals in their views on whether a woman can live a fulfilling life without children. Respondents without children are significantly more likely to accept of male voluntary childlessness than parents in both dimensions.

### Results for individual-level variables for items considering male voluntary childlessness

Estimates of the multilevel logistic regressions about attitudes towards male voluntary childlessness based on EVS data are shown in Tables 3 and 4.

**Table 3:** Results of the multilevel logistic regression predicting attitudes of male voluntary childlessness, ESS data 2018.

	Model A	Model B	Model C	Model D
Approve if a woman chooses not to have children				
*Male*	*1.000*	*1.000*	*1.000*	*1.000*
Female	1.134***	1.134***	1.134***	1.134***
18-30	1.315***	1.315***	1.315***	1.315***
31-45	1.526***	1.525***	1.527***	1.526***
46-60	1.324***	1.324***	1.325***	1.324**
>60	*1.000*	*1.000*	*1.000*	*1.000*
Low (ISCED 0-2)	0.964	0.963	0.964	0.964
*Medium (ISCED 3-4)*	*1.000*	*1.000*	*1.000*	*1.000*
High (ISCED 5-6)	1.110*	1.110*	1.109*	1.110*
*Paid job*	*1.000*	*1.000*	*1.000*	*1.000*
Not in paid job	0.953	0.953	0.952	0.953
Retired	0.861*	0.861*	0.861*	0.861*
At least once a week	0.493***	0.493***	0.494***	0.493***
At least once a month	0.635***	0.635***	0.636***	0.636***
Only on special holy days	0.717***	0.717***	0.717***	0.717***
Less often	0.781***	0.781***	0.781***	0.781***
*Never*	*1.000*	*1.000*	*1.000*	*1.000*
Single	1.184***	1.184***	1.184***	1.184***
Cohabiting	1.249***	1.249***	1.247***	1.249***
*Married*	*1.000*	*1.000*	*1.000*	*1.000*
*Yes, have children*	*1.000*	*1.000*	*1.000*	*1.000*
Not having children	1.404***	1.404***	1.404***	1.405***
CHILDLESSNESS		1.064		
GII			0.872***	
ATTENDANCE				1.262
Constant	0.434***	0.159**	1.708	0.214
Constant (country)	3.729***	3.201***	1.826***	3.637***
ll likelihood	-11608.9	-11607.3	-11598.5	-11608.8
Wald Chi2	710.5	713.9	774.3	711.4
N (individuals/countries)	21389/27			

The standard errors are adjusted for clustering at the country level. *  p < 05; ** p < .01; *** p < .001.

**Table 4:** Results of the multilevel logistic regression predicting attitudes of male voluntary childlessness, EVS data 2008.

	Model A	Model B	Model C	Model D
A man does not need children to be fulfilled				
*Male*	*1.000*	*1.000*	*1.000*	*1.000*
Female	1.507***	1.507***	1.507***	1.507***
18-30	1.008	1.008	1.008	1.007
31-45	1.298***	1.298***	1.299***	1.298***
46-60	1.239***	1.239***	1.239***	1.238***
>60	*1.000*	*1.000*	*1.000*	*1.000*
Low (ISCED 0-2)	0.825***	0.826***	0.826***	0.825***
Medium (ISCED 3-4)	*1.000*	*1.000*	*1.000*	*1.000*
High (ISCED 5-6)	1.147***	1.147***	1.147***	1.148***
*Paid job*	*1.000*	*1.000*	*1.000*	*1.000*
Not in paid job	0.957	0.957	0.957	0.957
Retired	0.943	0.943	0.943	0.943
At least once a week	0.608***	0.608***	0.609***	0.608***
At least once a month	0.632***	0.631***	0.632***	0.631***
Only on special holy days	0.739***	0.739***	0.739***	0.739***
Less often	0.859**	0.859**	0.859**	0.859**
*Never*	1	1	1	1
Single	1.052	1.052	1.052	1.052
Cohabiting	1.187***	1.187***	1.187***	1.187***
*Married*	*1.000*	*1.000*	*1.000*	*1.000*
*Yes, have children*	*1.000*	*1.000*	*1.000*	*1.000*
Not having children	1.815***	1.815***	1.815***	1.815***
CHILDLESSNESS		1.126*		
GII			0.907**	
ATTENDANCE				0.316
Constant	0.407***	0.114***	9.907***	1.836
Constant (country)	3.724***	2.957***	2.412***	3.268***
ll likelihood	-18296.3	-18294.1	-18291.3	-18295.2
Wald Chi2	1087.1	1092.2	1099.1	1089.8
N (individuals/countries)	34660/27			

The standard errors are adjusted for clustering at the country-level. *  p < 05; ** p < 0.01; *** p < 0.001.

Similar to attitudes towards female voluntary childlessness, we found that women have more accepting attitudes than men in both dimensions. In terms of age, those in the younger age groups are more accepting if a man chooses not to have children compared to those in the oldest age group. However, regarding proscriptive attitudes, the youngest age group (18-30) does not differ significantly from the oldest age group, but those aged between 31 and 60 are less likely to agree that a man needs to have children to be fulfilled.

As for educational level, we found a positive association between having a high level of education and the acceptance of voluntary male childlessness in both measures. However, low-educated respondents do not differ significantly from the reference category in the prescriptive dimension, but they are less likely to agree that men do not need children to be fulfilled compared to respondents with a medium level of education.

Regarding employment status, a significant association was found only for women: pensioners are less accepting of female voluntary childlessness in both dimensions. However, unlike for women, no significant difference was found for those not in paid work in the proscriptive dimension.

Religiosity and attitudes towards male voluntary childlessness showed significant differences in both dimensions, similar to the findings for women. Regarding partnership status, we obtained the same results as in the case of women: those who live in a cohabiting relationship are significantly more likely to approve of female voluntary childlessness than married respondents. While single individuals differed significantly from married ones only in the prescriptive dimension. Regarding parental status, we obtained the same result for both male and female voluntary childlessness: Respondents without children are significantly more likely to accept voluntary childlessness in both dimensions.

### Country-level variables

Regarding the country-level variables, there are significant differences between countries. For countries’ childlessness rates, we observed a significant association between proscriptive attitudes and countries’ childlessness rates. Specifically, higher childlessness rates predict higher acceptance levels of both voluntary female and male childlessness in the EVS data (model B). However, there is no significant association between the childlessness rate and attitudes towards voluntary childlessness in the prescriptive dimension (model B).

Furthermore, we found a significant association between the GII and attitudes towards voluntary childlessness. Higher gender inequality goes together with less acceptance of both female and male voluntary childlessness in both dimensions. The likelihood ratio test suggests that this is the best fitting model for both the ESS and EVS data (model C).

The aggregated attendance of religious services does not predict attitudes towards voluntary childlessness, neither the ESS nor the EVS models (model D). Hence, religiousness predicts attitudes towards voluntary childlessness only at the individual level.

### Robustness checks

To verify the robustness of the above results, we conducted several sensitivity analyses, with the most important ones outlined here. All findings can be found in the Supplementary Material. The first robustness analysis examines different measures of religiosity. As mentioned above, in the main analysis, we used church attendance as a dimension of religiosity, as it could be harmonized between the two databases. However, both databases also included other measures of religiosity. In the ESS database, we assessed subjective religiosity using the following variable: “Regardless of whether you belong to a particular religion, how religious would you say you are?” Respondents rated their religiosity on an 11-point scale, where 0 indicated “not at all religious” and 10 indicated “very religious.” This variable was treated as continuous in our analysis.

In the EVS, we identified the following item to measure subjective religiosity: “Independently of whether you go to church or not, would you say you are [a religious person, not a religious person, a convinced atheist]?” Given the very small number of respondents identifying as “convinced atheists,” we merged this category with “not a religious person” for the analysis.

The results from the models are consistent with the main findings, indicating that religiosity, in any form, has a significant effect at the individual level across all models. However, no significant associations were found at the macro level for any measure (See S3 and S4 Tables). Based on these findings, we can hypothesize that religiosity influences attitudes toward childlessness only at the individual level.

The second robustness analysis was conducted to determine whether we would obtain different results using the ESS 2006 database instead of ESS 2018. The results show very similar patterns, indicating that the same factors are associated with attitudes toward voluntary childlessness in both 2006 and 2018. Only slight differences were identified: in the male model, gender and education were not significantly associated with male voluntary attitudes. However, the macro level indicators were associated in the models as in the models of 2018. Since we could not find the GII value for 2006, we used the 2008 value in the analysis. The detailed results are provided in the appendices (See S5 and S6 Tables).

## Discussion and conclusion

Exploring social attitudes towards female and male voluntary childlessness is an underrepresented field of social scientific analysis, especially from a comparative perspective. This may be due to the lack of a clear definition of the concept of voluntary childlessness. There are many situations where individuals might want to have children, but circumstances (such as partnership stability, financial situation, or health issues) prevent this from happening. This fluidity has been pointed out in several studies [16,30,39,40]. Since the concept of voluntary childlessness is not clearly defined, it also remains unclear how to measure it. Although international surveys have begun to ask questions about attitudes towards voluntary childlessness, there is still uncertainty about how to measure it effectively and what the current indicators capture. A more realistic approach might involve specifying a set of questions that better reflect the different dimensions of voluntary childlessness.

To shed more light thereon, we analysed two international social surveys, namely the European Social Survey and the European Values Studies, both of which include items on female and male voluntary childlessness. In the ESS, the item focuses on the prescriptive dimension of voluntary childlessness, i.e. it stresses the social expectation of parenthood. This item is formulated to focus on active decision-making; however, it is not clear how respondents understand and interpret this question. A Dutch national survey entitled Cultural Change in the Netherlands was clearer when it used almost the same item, but they added the half-sentence circumscribing *‘A married couple decides to not have children, while there are no medical restrictions’* [8]. The ESS item does not filter out active decision-making when somebody chooses not to have children because of health-related concerns or the lack of a partner. Thus, we suggest survey designers to complete the item with this clarification.

The EVS item focuses on the proscriptive dimension of voluntary childlessness, which means that it stresses the consequences of not having children. This item does not exclude those who cannot have children due to medical reasons either; however, this is less significant because the item does not focus on active decision-making.

Our first aim was to explore whether these two items can equally measure the attitude towards voluntary childlessness by examining the same socio-demographic and family related factors. A second aim was to examine if and to what extent different country level indicators predict attitudes towards the two dimensions of voluntary childlessness. To do so, we included the same countries and the same independent variables. We interpret our results within the frameworks of the New Home Economics and Gender Revolution theories and the Second Demographic Transition.

Women tend to be more accepting of childlessness, which may reflect not only their awareness of the potential opportunity costs related to labour market disruptions due to childbearing, as suggested by the New Home Economics framework [27], but also their greater awareness of the physical, emotional, and psychological costs of childbearing and childrearing—costs that they bear to a much larger extent than men. This heightened awareness could contribute to their higher tolerance of the decision not to have a child. Additionally, individuals with higher levels of education are more accepting of both female and male voluntary childlessness in both dimensions, which is consistent with the New Home Economics literature [23], as they face higher opportunity costs related to childbearing. Employment status plays a role only in the prescriptive dimension, with retirees being less accepting of both male and female voluntary childlessness.

Regarding the Gender Inequality Index at the country level, our findings indicate its significant role in predicting attitudes towards both male and female voluntary childlessness in both dimensions. In societies with low gender inequality, women often play a larger role in economic stability and are seen in roles beyond motherhood, which aligns with the research of Rijken and Merz [11] and the Gender Revolution theory.

According to the Second Demographic Transition theory, younger, higher-educated, and less religious individuals are expected to be more accepting of childlessness compared to their older, lower-educated, and more religious counterparts. Our results support this expectation in both dimensions, where younger respondents and women show more favourable attitudes towards both female and male voluntary childlessness. This is consistent with previous empirical findings [11,15,21,32]. Additionally, religiosity plays a significant role; non-religious individuals are more accepting of new family types, including childless families [21,22,32]. Our results reinforce this: increased attendance at religious services correlates with less acceptance of both dimensions of voluntary childlessness. Conversely, country-level religiosity does not seem to influence attitudes towards voluntary childlessness, which is a novel contribution to the literature, as prior studies have not explored this relationship.

We also controlled for relationship status and whether someone has children. Those who live in cohabitation have more favourable attitudes towards both male and female childlessness in both dimensions, which is consistent with previous empirical findings [11,32]. However, singles show more favourable attitudes only in the prescriptive dimension towards the acceptance of male and female voluntary childlessness, but not in the proscriptive dimension. Additionally, having children significantly predicts that individuals are less accepting of both male and female voluntary childlessness in both dimensions.

We found that the childlessness rate significantly associates with the proscriptive dimension. In countries with a higher childlessness rate, respondents are less likely to agree with the statement that men and women cannot lead a fulfilling life without children. However, the prevalence of childlessness does not significantly associate with agreement on the statement that men or women can choose not to have children.

Analysing attitudes towards voluntary childlessness is important in the future for several reasons. First, many former socialist countries have pronatalist policy programmes [41,42], which leads to higher social opposition to remaining childfree by choice in Central and Eastern European countries compared to Western European countries [10,11]. Our descriptive findings also confirmed this statement: Central Eastern European countries stand out from Western European continental countries, particularly Northern European countries, in their acceptance of voluntary childlessness across both dimensions. Furthermore, we must also consider that there is a growing trend in Central and Eastern Europe towards family policies that are more interventionist and may create disadvantaged positions for those who remain childless [19,42]. Second, a recent study revealed that childless people are more likely to vote differently than their parents [43] and it is likely that individuals who have more favourable attitudes towards voluntary childlessness also have different political views and behaviours. Third, as the number of voluntarily childless individuals will increase, for example, due to young people choosing to not have their own biological children because they are worried about overpopulation and/or climate change [44], it is important to include these questions in international databases. Shedding more light on how to properly measure voluntary childlessness is crucial for efficient family policymaking.

It is extremely relevant to understand what people have in mind when they are surveyed about voluntary childlessness. We found no difference in attitudes towards female and male voluntary childlessness; this indicates that how and in which dimensions attitudes towards voluntary childlessness are measured is more important than whether the question applies to men or women. Furthermore, in the future, it is worthwhile to pay attention to those who do not have a biological child but have a social and emotional relationship with a child. They are considered childless by some but not by others, and this distinction likely varies across European societies. Qualitative studies should examine the concepts people have in mind to strengthen our understanding of international comparisons.

## Supporting information

S1 Fig Relationship between the proportion of respondents who choose “a woman does not need a child to be fulfilled” (EVS) and the proportion of respondents who approve if “a woman chooses never to have children” (ESS) in 27 European countries (%). Source: ESS data 2018 and EVS data 2008. The figure shows the proportion of respondents by country who strongly agree and agree with the ESS item, and who believe that a woman does not necessarily need a child to be fulfilled for the EVS item. A strong correlation can be observed among the countries. The Northern European countries are more accepting in both dimensions, while the Central and Eastern European countries are less accepting in both dimensions.
(PDF)

S2 Fig Relationship between the proportion of respondents who choose “a man does not need a child to be fulfilled” (EVS) and proportion of respondents who approve if “a man chooses never to have children” (ESS) in 27 European countries. Source: ESS data 2018 and EVS data 2008. The figure shows the proportion of respondents by country who strongly agree and agree with the ESS item, and who believe that men do not need a child to be fulfilled. In this case as well, the majority of those who do not accept voluntary childlessness are found in the Central and Eastern European countries.
(PDF)

S1 Table The included database and variables. (PDF)

S2 Table Descriptive statistics for variables included in the analysis. Source: European Value Study 2008 dataset and European Social Survey 2018 dataset.
(PDF)

S3 Table Results of the multilevel logistic regression: predicting attitudes towards female and male voluntary childlessness in models A and D using different measurements of religiosity, ESS data 2018. (PDF)

S3 Table Results of the multilevel logistic regression: predicting attitudes towards female and male voluntary childlessness in models A and D using different measurements of religiosity, EVS data 2008. (PDF)

S5 Table Results of the multilevel logistic regression: predicting attitudes of female voluntary childlessness, ESS data 2006. (PDF)

S6 Table Results of the multilevel logistic regression: predicting attitudes of male voluntary childlessness, ESS data 2006. (PDF)
