# Supplementary material for: Measuring attitudes towards voluntary childlessness: Indicators in European comparative surveys
Source: PLoS One. 2025 Mar 19;20(3):e0319081. doi: 10.1371/journal.pone.0319081 (PMC11922256; doi:10.1371/journal.pone.0319081)
Supplement: S1 Fig — Source: ESS data 2018 and EVS data 2008. The figure shows the proportion of respondents by country who strongly agree and agree with the ESS item, and who believe that a woman does not necessarily need a child to be fulfilled for the EVS item. A strong correlation can be observed among the countries. The Northern European countries are more accepting in both dimensions, while the Central and Eastern European countries are less accepting in both dimensions. (PDF) [file pone.0319081.s001.pdf]

S1 Figure

Relationship between the proportion of respondents who choose "a woman does not need a child to be fulfilled" (EVS) and the proportion of respondents who approve if "a woman chooses never to have children" (ESS) in 27 European countries

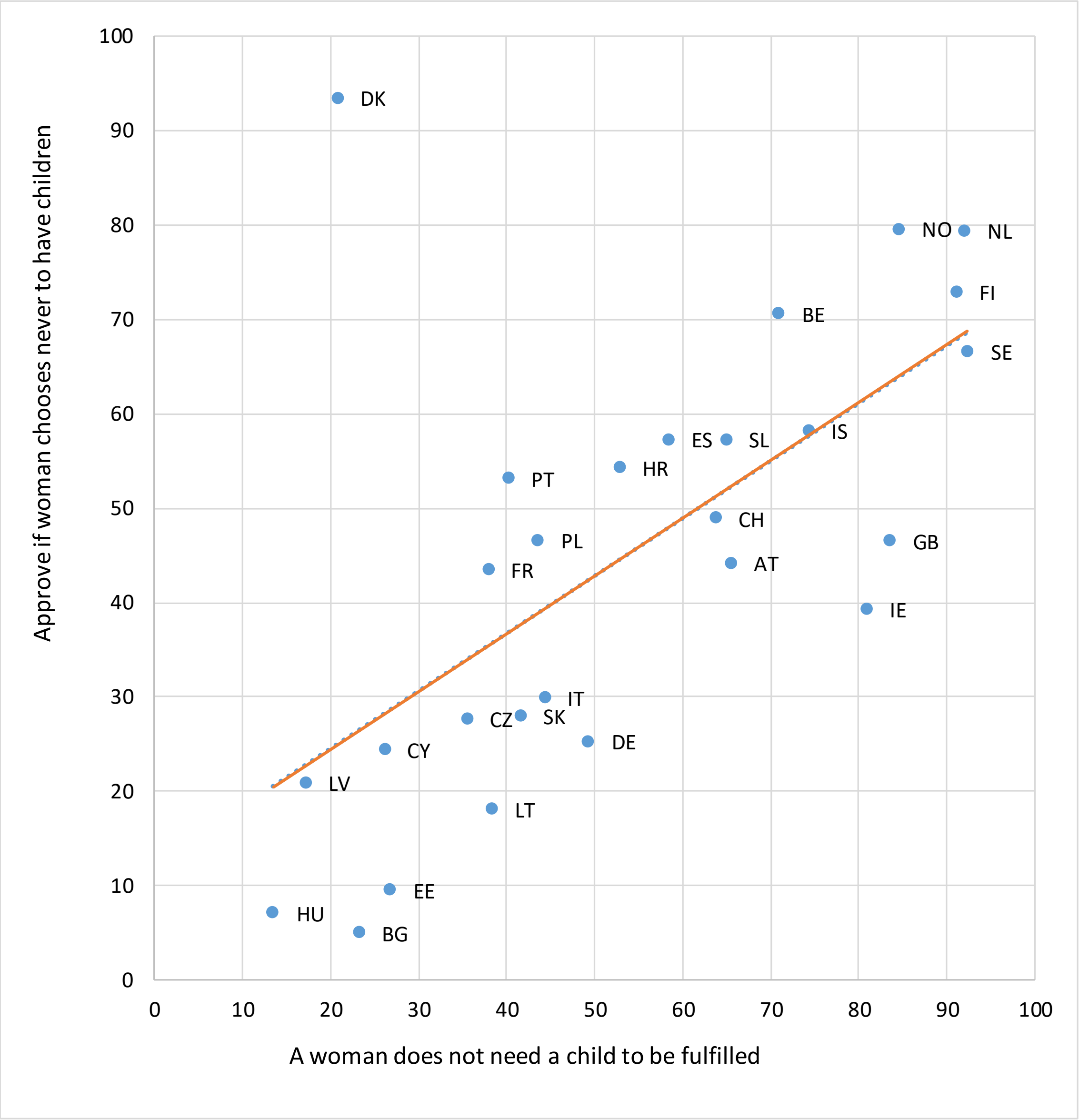

Source: ESS data 2018 and EVS data 2008

Note: The answer option strongly agrees and agrees for the ESS item and not necessarily need a child for a woman to be fulfilled for the EVS item.
