# Supplementary material for: Measuring attitudes towards voluntary childlessness: Indicators in European comparative surveys
Source: PLoS One. 2025 Mar 19;20(3):e0319081. doi: 10.1371/journal.pone.0319081 (PMC11922256; doi:10.1371/journal.pone.0319081)
Supplement: S2 Fig — Source: ESS data 2018 and EVS data 2008. The figure shows the proportion of respondents by country who strongly agree and agree with the ESS item, and who believe that men do not need a child to be fulfilled. In this case as well, the majority of those who do not accept voluntary childlessness are found in the Central and Eastern European countries. (PDF) [file pone.0319081.s002.pdf]

S2 Figure

Relationship between the proportion of respondents who choose "a man does not need a child to be fulfilled" (EVS) and proportion of respondents who approve if "a man chooses never to have children" (ESS) in 27 European countries

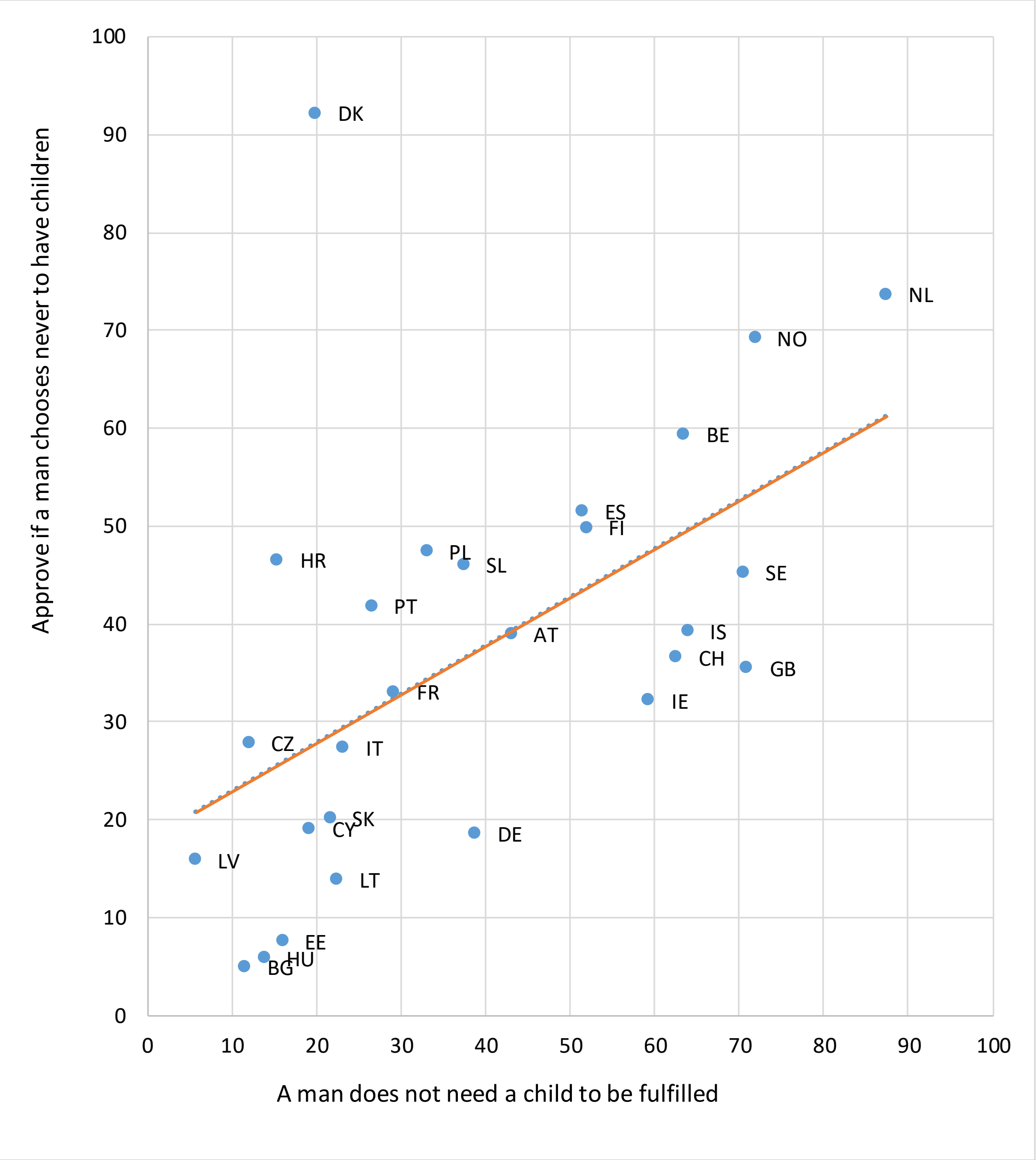

Source: ESS data 2018 and EVS data 2008

Note: The answer option strongly agrees and agrees for the ESS item and disagree strongly for the EVS item.
