# Supplementary material for: Measuring attitudes towards voluntary childlessness: Indicators in European comparative surveys
Source: PLoS One. 2025 Mar 19;20(3):e0319081. doi: 10.1371/journal.pone.0319081 (PMC11922256; doi:10.1371/journal.pone.0319081)
Supplement: S1 Table — (PDF) [file pone.0319081.s003.pdf]

S1 Table

The included database and variables

Data collection	9th round of the European Social Survey (ESS), conducted in 2018	4th round of the European Social Values (EVS), conducted in 2008
**Dependent variables about women**	How much do you approve or disapprove if a woman chooses never to have children?<br>Answer options:<br>1. approve strongly<br>2. approve<br>3. neither approve nor disapprove<br>4. disapprove<br>5. disapprove strongly	Do you think that a woman has to have children in order to be fulfilled or is this not necessary?<br>Answer options:<br>1 – needs children<br>2 – not necessary
**Dependent variables about men**	How much do you approve or disapprove if a man chooses never to have children?<br>Answer options:<br>1. approve strongly<br>2. approve<br>3. neither approve nor disapprove<br>4. disapprove<br>5. disapprove strongly	Do you think that a man has to have children in order to be fulfilled or is this not necessary?<br>Answer options:<br>1. agree strongly<br>2. agree<br>3. neither agree nor disagree<br>4. disagree<br>5. disagree strongly
**Individual-level covariates:**	Socio-demographics: sex, age group, education, employment, religiosity, marital/partnership status, parenthood status	
**Macro-level factors:**	Childlessness rate, GII, Aggregate mean at country level of attendance of religious services	
