# Supplementary material for: Measuring attitudes towards voluntary childlessness: Indicators in European comparative surveys
Source: PLoS One. 2025 Mar 19;20(3):e0319081. doi: 10.1371/journal.pone.0319081 (PMC11922256; doi:10.1371/journal.pone.0319081)
Supplement: S2 Table — Source: European Value Study 2008 dataset and European Social Survey 2018 dataset. (PDF) [file pone.0319081.s004.pdf]

S2 Table
Descriptive statistics for variables included in the analysis

	ESS			EVS		
**Variable**	**Category range**	**N**	**%**	**Category range**	**N**	**%**
**Gender**	Male	19991	46.1	Male	15102	43.6
	Female	23352	53.9	Female	19558	56.7
**Age groups**	18-30	6657	15.3	18-30	6801	19.6
	31-45	9791	22.6	31-45	8886	25.6
	45-60	11633	26.8	46-60	9233	26.6
	>60	15262	35.2	>60	9740	28.1
**Education level**	Lower than secondary education	17140	39.6	Lower than secondary education	10401	30
	Secondary education	15207	35.1	Secondary education	16011	46.2
	Tertiary education	10996	25.4	Tertiary education	8253	23.8
**Employment status**	Employed	22667	52.3	Employed	18949	54.7
	Retired	8607	19.9	Retired	8692	25.1
	Not in paid job	12069	27.8	Not in paid job	7019	20.2
**Partnership status**	Married	17184	39.7	Married	18117	52.3
	Single	5605	12.9	Single	12949	37.3
	Having cohabiting partner	20554	47.4	Having cohabiting partner	3594	10.4
**Having children**	Yes	31223	72	Yes	25523	73.6
	No	12120	28	No	9142	26.4
**Attendance at religious services**	At least once a week	5750	13.3	At least once a week	5542	16
	At least once a month	4142	9.6	At least once a month	3051	10.1
	Only on special holy days	9654	22.3	Only on special holy days	7066	20.4

	Less often	8450	19.5	Less often	2671	7.7
	Never	15347	35.4	Never	15880	45.8
Childlessness rate	Continuous	min= 5.8%<br>max= 23.7%<br>mean=12.3%<br>standard deviation=4.6%		Continuous	min= 2.8%<br>max= 21.9%<br>mean=10.7%<br>standard deviation=3.9%	
GII	Continuous	min=0.004<br>max=0.258<br>mean=0.09<br>standard deviation=0.06		Continuous	min=0.174<br>max=0.409<br>mean=0.29<br>standard deviation=0.06	
ATTENDANCE religious services	Continuous	min=1.06<br>max=3.22<br>mean=2.53<br>standard deviation=0.52		Continuous	min=0.7<br>max=1.79<br>mean=1.18<br>standard deviation=0.29s	

Source: European Value Study 2008 dataset and European Social Survey 2018 dataset
