# Supplementary material for: Measuring attitudes towards voluntary childlessness: Indicators in European comparative surveys
Source: PLoS One. 2025 Mar 19;20(3):e0319081. doi: 10.1371/journal.pone.0319081 (PMC11922256; doi:10.1371/journal.pone.0319081)
Supplement: S3 Table — (PDF) [file pone.0319081.s005.pdf]

Table S3. Table Results of the multilevel logistic regression: predicting attitudes towards female and male voluntary childlessness in models A and D using different measurements of religiosity, ESS data 2018

	**Model A**	**Model D**	**Model A**	**Model D**
	**Approve if woman chooses not to have children**		**Approve if man chooses not to have children**	
Male	*1.000*	*1.000*	*1.000*	*1.000*
Female	1.311***	1.312***	1.312***	1.311***
18-30	1.237**	1.237**	1.238**	1.237**
31-45	1.399***	1.399***	1.401***	1.400***
46-60	1.279***	1.279***	1.280***	1.279***
>60	*1.000*	*1.000*	*1.000*	*1.000*
Low (ISCED 0-2)	0.905**	0.904**	0.904**	0.904**
Medium (ISCED 3-4)	*1.000*	*1.000*	*1.000*	*1.000*
High (ISCED 5-6)	1.171***	1.171***	1.170***	1.171***
Paid job	*1.000*	*1.000*	*1.000*	*1.000*
Not in paid job	0.901*	0.901*	0.901*	0.901*
Retired	0.830**	0.830**	0.831**	0.830**
Religious				
Single	1.135**	1.135**	1.135**	1.135**
Cohabiting	1.248***	1.249***	1.247***	1.248***
Married	*1.000*	*1.000*	*1.000*	*1.000*
Yes, have children	*1.000*	*1.000*	*1.000*	*1.000*
Not having children	1.478***	1.478***	1.479***	1.479***
RELIGIOUSITY		1.262		1.262
Constant	0.633	0.214*	2.968***	0.228
Constant (country)	4.523***	3.786***	1.824***	4.294***
ll likelihood	-12070.3	-12070.2	-11599.7	-11599.7
Wald Chi2	814.7	814.8	730.3	730.4
N (individuals/countries)	21954/27		21389/27	

The standard errors are adjusted for clustering at the country-level. * p<05; ** p<.01; *** p<.001
