# Supplementary material for: Measuring attitudes towards voluntary childlessness: Indicators in European comparative surveys
Source: PLoS One. 2025 Mar 19;20(3):e0319081. doi: 10.1371/journal.pone.0319081 (PMC11922256; doi:10.1371/journal.pone.0319081)
Supplement: S3 Table — (PDF) [file pone.0319081.s006.pdf]

S4. Table Results of the multilevel logistic regression: predicting attitudes towards female and male voluntary childlessness in models A and D using different measurements of religiosity, EVS data 2008

	Model A	Model D	Model A	Model D
	**A woman does not need children to be fulfilled**		**A man does not need children to be fulfilled**	
Male	*1.000*	*1.000*	*1.000*	*1.000*
Female	1.243***	1.243***	1.243***	1.243***
18-30	1.210**	1.210**	1.210**	1.209**
31-45	1.516***	1.516***	1.516***	1.515***
46-60	1.384***	1.384***	1.384***	1.383***
>60	*1.000*	*1.000*	*1.000*	*1.000*
Low (ISCED 0-2)	0.783***	0.783***	0.783***	0.783***
Medium (ISCED 3-4)	*1.000*	*1.000*	*1.000*	*1.000*
High (ISCED 5-6)	1.134***	1.134***	1.134***	1.134***
Paid job	*1.000*	*1.000*	*1.000*	*1.000*
Not in paid job	0.939	0.939	0.939	0.939
Retired	0.921	0.921	0.922	0.921
Religious	*1*	*1*	*1*	*1*
Not religious				
Single	1.006	1.006	1.006	1.006
Cohabiting	1.117*	1.117*	1117*	1.117*
Married	*1.000*	*1.000*	*1.000*	*1.000*
Yes, have children	*1.000*	*1.000*	*1.000*	*1.000*
Not having children	2.217***	2.217***	2.216***	2.217***
RELIGIOUSITY				0.316
Constant	0.944	0.329	17.697**	2.494
Constant (country)	4.653***	3.972	3.229***	4.405***
ll likelihood	-19003.522	-19002.5	-18334.427	-18333.6
Wald Chi2	1573.31	1575.1	1018.52	1019.9
N (individuals/countries)	34660/27		34660/27	

The standard errors are adjusted for clustering at the country-level. * p<05; ** p<.01; *** p<.001
