# Supplementary material for: Measuring attitudes towards voluntary childlessness: Indicators in European comparative surveys
Source: PLoS One. 2025 Mar 19;20(3):e0319081. doi: 10.1371/journal.pone.0319081 (PMC11922256; doi:10.1371/journal.pone.0319081)
Supplement: S5 Table — (PDF) [file pone.0319081.s007.pdf]

S5 Table. Results of the multilevel logistic regression: predicting attitudes of female voluntary childlessness, ESS data 2006

	Model A	Model B	Model C	Model D
Approve if woman chooses not to have children				
Male	*1.000*	*1.000*	*1.000*	*1.000*
Female	1.266***	1.266***	1.266***	1.266***
18-30	1.036	1.038	1.038	1.037
31-45	1.336***	1.337***	1.338***	1.337***
46-60	1.305***	1.306***	1.307***	1.306***
>60	*1.000*	*1.000*	*1.000*	*1.000*
Low (ISCED 0-2)	0.811***	0.811***	0.812***	0.811***
Medium (ISCED 3-4)	*1.000*	*1.000*	*1.000*	*1.000*
High (ISCED 5-6)	1.059	1.058	1.058	1.059
Paid job	*1.000*	*1.000*	*1.000*	*1.000*
Not in paid job	0.947	0.947	0.947	0.947
Retired	0.866	0.867	0.868	0.867
At least once a week	0.476***	0.476***	0.476***	0.477***
At least once a month	0.605***	0.605***	0.605***	0.606***
Only on special holy days	0.712***	0.712***	0.711***	0.712***
Less often	0.792***	0.792***	0.792***	0.792***
Never	*1.000*	*1.000*	*1.000*	*1.000*
Single	1.027	1.027	1.027	1.027
Cohabiting	1.254**	1.253**	1.251**	1.253**
Married	*1.000*	*1.000*	*1.000*	*1.000*
Yes, have children	*1.000*	*1.000*	*1.000*	*1.000*
Not having children	1.592***	1.591***	1.591***	1.592***
CHILDLESSNESS		1.171		
GII			0.858***	
ATTENDANCE				1.688
Constant	0.373**	0.072**	1.456	0.106

Constant (country)	6.268**	4.779**	2.561**	5.691**
ll likelihood	**-9017.5**	**-9015.8**	**-9010.6**	**-9016.9**
Wald Chi2	**523.3**	**526.8**	**541.9**	**524.3**
N (individuals/countries)	**17879/21**			

The standard errors are adjusted for clustering at the country-level. * p<05; ** p<.01; *** p<.001
