# Supplementary material for: Measuring attitudes towards voluntary childlessness: Indicators in European comparative surveys
Source: PLoS One. 2025 Mar 19;20(3):e0319081. doi: 10.1371/journal.pone.0319081 (PMC11922256; doi:10.1371/journal.pone.0319081)
Supplement: S6 Table — (PDF) [file pone.0319081.s008.pdf]

S6 Table. Results of the multilevel logistic regression: predicting attitudes of male voluntary childlessness, ESS data 2006

	Model A	Model B	Model C	Model D
Approve if a man chooses not to have children				
Male	*1.000*	*1.000*	*1.000*	*1.000*
Female	1.003	1.003	1.003	1.002
18-30	1.121	1.122	1.123	1.121
31-45	1.576***	1.577***	1.578***	1.577***
46-60	1.267***	1.268**	1.269**	1.267**
>60	*1.000*	*1.000*	*1.000*	*1.000*
Low (ISCED 0-2)	0.841***	0.841***	0.841***	0.841***
Medium (ISCED 3-4)	*1.000*	*1.000*	*1.000*	*1.000*
High (ISCED 5-6)	1.071	1.071	1.071	1.071
Paid job	*1.000*	*1.000*	*1.000*	*1.000*
Not in paid job	0.971	0.971	0.972	0.971
Retired	0.894	0.894	0.895	0.894
At least once a week	0.519***	0.519***	0.520***	0.519***
At least once a month	0.570***	0.571***	0.570***	0.571***
Only on special holy days	0.676***	0.677***	0.676***	0.676***
Less often	0.845**	0.845**	0.845**	0.845**
Never	*1.000*	*1.000*	*1.000*	*1.000*
Single	1.074	1.074	1.074	1.074
Cohabiting	1.081	1.081	1.079	1.081
Married	*1.000*	*1.000*	*1.000*	*1.000*
Yes, have children	*1.000*	*1.000*	*1.000*	*1.000*
Not having children	1.620***	1.619***	1.619***	1.620***
CHILDLESSNESS		1.137		
GII			0.864***	
ATTENDANCE				1.407
Constant	0.298***	0.077**	1.095	0.131

Constant (country)	5.325**	4.449**	2.356**	5.108**
ll likelihood	**-8506.9**	**-8505.7**	**-8500.1**	**-8506.7**
Wald Chi2	**510.1**	**512.5**	**528.5**	**510.6**
N (individuals/countries)	**17732/21**			

The standard errors are adjusted for clustering at the country-level. * p<05; ** p<.01; *** p<.001
